# Forecasting Food Innovations with a Delphi Study

**DOI:** 10.3390/foods11223723

**Published:** 2022-11-19

**Authors:** Alexis Zickafoose, Peng Lu, Mathew Baker

**Affiliations:** Department of Agricultural Leadership, Education, and Communications, Texas A&M University, College Station, TX 77843, USA

**Keywords:** novel food, development, health, functional food, novel technology, qualitative study

## Abstract

Food innovations can create novel nutritious food, improve agricultural sustainability, and increase the agri-food industry’s market profits. Our study proposes a consensus definition of food innovations and forecasts food innovations that will be available to consumers in the next five years by using a Delphi study. Thirteen experts aged 35 to 85 from the US and the UK researching or working in agriculture and nutrition, public health, the agri-food industry, or food policy participated in three rounds of this Delphi study. The experts were chosen using the snowball sampling method. This study followed the implementation and data analysis guidelines popularized by the Rand Corporation. The consensus definition for food innovations (with 76.9% agreement) was that ‘food innovations aid in the development, production, or transportation of new food products, processes, or technology to promote human health, food security, or environmental sustainability’. The specific food innovations, which had over 69% agreement, are ranked as (1) plant-based meat alternatives, (2) personalized nutrition, (3) natural foods, (4) new genetically modified organisms, (5) regenerative agriculture, (6) urban agriculture, (7) packing innovations, (8) alternative flours, (9) improving shelf life, (10) supply chain technologies, (11) improved soil health, and (12) technology for traceability. The food innovation definition and identified specific food innovations could further connect the agricultural value chain to develop novel nutritious foods and improve agricultural sustainability. Agri-food industry specialists, practitioners, researchers, and policymakers can advance food innovation development and research pinpointing the specific food innovations along the agricultural value chain.

## 1. Introduction

The advancement of science and technologies introduces a variety of food innovations. Research institutions and the food industry are looking for ways to produce food that promotes health, increases food security, and achieves environmental sustainability. Novel technologies such as 3D printing, pulse electric field, and the combination of novel technologies with traditional methods are being developed to increase food quality and nutritional value [1]. The European Union (EU) defines novel foods as those which did not exist or were created by processes that did not exist in the EU prior to 15 May 1997 [2]. While the EU has an accepted definition, to the best of our knowledge, the United States lacks a unified definition for novel foods or food innovations.

Despite the existence of the numerous food innovations emerging throughout the agricultural value chain, the trends of upcoming food innovations remain unclear. In addition, few food innovations have been comprehensively investigated in the food industry for consumer adoption [3]. Food innovations are essential for food industry firms to increase their market profits in a competitive environment. The growth of food innovations positively affects the agri-food industry’s profits. Therefore, it is necessary for food industry companies to comprehend the trends and changing dynamics of upcoming food innovations in the global food sector in order to decrease their economic inputs. However, many agri-food companies have been going through a high failure rate of launching new products as these products did not meet consumers’ expectations [4]. Identifying emerging food innovations is critical for successfully launching food innovations, assessing consumer acceptance, and increasing economic growth.

Food innovations have the potential to feed the growing global population, improve public health and benefit environmental sustainability [5]. Providing adequate healthy and nutritious food as a way of supporting environmental outcomes requires great efforts from food system stakeholders. Although the food system delivers food calories to keep pace with the world’s population, more than 820 million people have inadequate food, and many people cannot access high-quality diets [5]. Meanwhile, the food system has been facing the challenges of reducing negative impacts on environmental conditions [6]. Thus, the development, production, and transportation of new food products or technology can contribute to the solutions for addressing global food insecurity, health problems, and environmental issues.

One of the primary solutions to increasing food production yield is using innovative technologies. An example of innovative food technology is a new frontier of genome engineering called gene-editing technology. A variety of crop yields (e.g., rice, wheat, maize) have been increased by using gene-editing technology [7]. Moreover, other emerging innovative technologies, such as precision farming, artificial intelligence, and biological-based crop protection, have been investigated to increase crop yields [8]. In addition, given that many people are suffering from inadequate nutritional intake and many environmental conditions are negatively influenced by food systems, numerous food technologies have emerged with potential applications to address these issues. For example, a broad set of novel food technologies (e.g., nanotechnology, food irradiation, gene technology, and cultured meat) related to food production, preparation, and transportation have been introduced into the food supply chain as part of the solutions for enhancing environmental sustainability [9]. To accelerate the innovative food products and technologies development and adoption, it is crucial for researchers to provide empirical scientific information for food system stakeholders.

Our study can guide future research on upcoming food innovations and their adoption by consumers, institutions, and food industry companies. The identified food innovations can build the bridge between food science and society. Specifically, food industry experts, researchers, and policymakers can use the identified food innovations to advance novel food product development and research. In addition, further investigating consumers’ perceptions on the upcoming food innovations can better facilitate the food innovation adoption process seeing as consumer characteristics are essential drivers of adoption [10].

### The Delphi Method Background

The Delphi method was developed by the RAND Corporation to forecast technological advancements and social developments [11,12,13,14,15,16,17]. Since its inception, it has been used by a variety of disciplines to produce and develop research [18,19]. The Delphi method provides a structured process for collecting and synthesizing knowledge from a group of experts through sequential questionnaires created from feedback and opinions obtained from prior responses [11,19]. It is a well-established method for building consensus among individuals with expertise in a particular topic [20]. It works on three principles: anonymity, iteration, and feedback [21]. The experts are approached online and are kept anonymous from one another, which encourages individuals with minority opinions to present their views [21,22]. A Delphi study is conducted in at least two rounds. Round one is traditionally the exploratory round which aims to gather information from the panel of experts on the forecast; the subsequent rounds ask the participants to provide quantitative estimates [11,21]. The Delphi technique works by using data provided by the panel and giving it back to them until an a priori consensus is reached [21]. Consensus building is one of the five objectives of a Delphi process [11]. The Delphi method prevents the opinion of one individual from overpowering the group by limiting social interactions [23]. In addition, the group projections applied in the Delphi process are more accurate than individual opinions [21]. Furthermore, obtaining opinions from a group of experts rather than amateurs can reduce cognitive biases and increase forecast accuracy [24,25,26,27,28,29]. The Delphi method’s accuracy is slightly increased over the number of rounds [21].

The Delphi technique aims to explore a likely outcome or reliably establish new structures rather than generalizing results [21,30]. The panel composition should “represent the synthesis of opinion of the particular group, no more, no less” [31] (p.4). If the identified experts have prolonged and constant experience in the topic, then 10 to 15 panelists is deemed sufficient; some even suggest that seven panelists are adequate [11,19,32]. Other research suggests that the more experts present, the more accurate the results; however, a causal connection cannot be established on this relationship [31]. There is no consensus in the literature on the ideal number of experts for the panel in a Delphi study.

This Delphi study aims to (1) generate a consensus among experts on a definition of food innovations and (2) forecast an overview of food innovations that are likely to be available to consumers in the next five years. The paper is structured as follows: in the next section the Delphi method is examined more closely. Then, the conduct of this study is presented. Subsequently, the results of the consensus definition and descriptive statistics on the forecasted food innovations are presented. The discussion of findings and the conclusion complete this article.

## 2. Materials and Methods

### 2.1. This Delphi Study Approach

A three-stage exploratory Delphi study was conducted to synthesize the current food innovation trends and generate a definition of food innovations. The study was conducted from November 2021 to February 2022. The methodology of [11] was used to guide the implementation and data analysis. Three rounds were conducted in succession. The first round was open-ended, allowing the experts to use their knowledge to propose a definition and forecast food innovations. The second round aimed to converge opinions; and the third round built consensus. A Delphi study panel comprises purposively selected experts who are knowledgeable in the field of the study’s research focus [21].

### 2.2. Participants

The inclusion criteria for selecting participants were based on whether the individual: was considered by their peers as an expert in agriculture and nutrition research, the food industry, public health, or food policy; held a position of authority across the aforementioned disciplines; and was knowledgeable about upcoming food innovations. Initially, 10 experts were identified purposively based on their positions in academia, government, and private industry and on the basis that they would have information on current food innovation research. They were invited to nominate the other experts for our Delphi study. They were asked to nominate themselves, since they fit the aforementioned criteria, or others they determined fit the inclusion criteria. Those nominated experts were then vetted by the researchers based on their experience and position in academia, government, and private industry.

In this study, 61 experts that fit the inclusion criteria were contacted using the snowball sampling method. The experts were from both the private and public sector. Of the 61 potential experts, 13 experts were willing and able to participate in each round of the Delphi study. The composition of the expert panel was mostly male (70%). Their fields include food policy (*n* = 4), animal science (*n* = 1), agricultural communications (*n* = 2), the poultry (*n* = 1) and beef (*n* = 1) industries, soil and crop sciences (*n* = 3), natural resources (*n* = 1), food technology (*n* = 1), and public health (*n* = 1). The participants primarily work in the United States (*n* = 12) and United Kingdom (*n* = 1). The composition of the panel has the interdisciplinary nature of the agricultural value chain and a possibility of food innovations across disciplines. Some of the participants are multidisciplinary and have expertise on several pertinent food- or agriculture-related subjects. There was no demographic data collected other than their research experience and field. The three-round Delphi study was conducted through Qualtrics. This Delphi study included an information sheet to provide informed consent of the participants per IRB guidelines. This was provided to prospective participants during the sampling process and when the first questionnaire was sent. By taking the questionnaire, it was understood that the experts consented to participate in the study.

**Figure 1:**
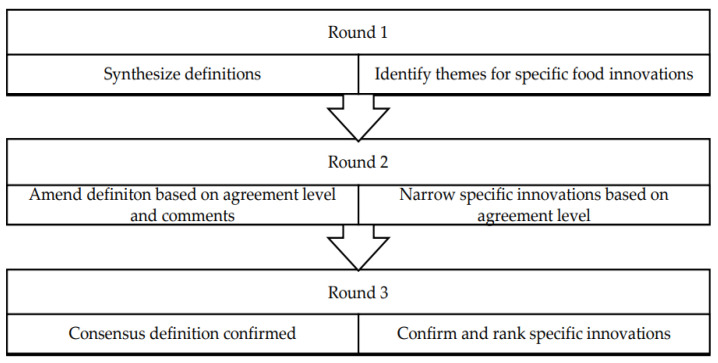
Methodology for the Delphi Study.

The 13 experts were given two weeks to complete each questionnaire. The participants received reminder emails with one week left to respond and two days left to respond to encourage completion. Thirteen experts (*n* = 13) responded to round one within two weeks. Through three rounds of questioning, the experts were enabled to reach a consensus on the food innovations definition and identify specific food innovations which are likely to be available to consumers by 2027 (Figure 1). Prior to each round, a trial run was conducted to minimize the risk of technical issues or misinterpretation of agreement statements.

### 2.3. Round One

The first round was exploratory in nature and provided the basis for questions to pose in the subsequent rounds. The experts were asked to provide a definition of food innovations based on their perspectives and a list of up to ten distinct food innovations that were most likely to be available to consumers within the next five years. Two independent researchers coded the data according to [11]. First, we classified the sentence fragments of food innovation definitions provided by the experts into three themes: how food innovations develop, what they are used for, and their implications. Repeated sentence fragments were eliminated during the open coding process. We synthesized three possible food innovation definitions based on the participant provided sentence fragments. Disagreements were solved by consulting the third author. In addition, we developed an amalgamated list of specific food innovations identified by the experts.

### 2.4. Round Two

The second round aimed to converge opinions on the food innovation definition and specific food innovations. The synthesized definitions and specific food innovations identified by the participants from the first round were used to create the round-two questionnaire. To ensure clarity, the specific food innovations were listed on the questionnaire. Following the methodology of [11], the researchers requested that the participants select their agreement level for the definitions and leave comments on the definitions. The participants were also asked to rate the likelihood of consumer availability for the distinct food innovations in the next five years on a 5-point Likert scale (1 = strongly disagree to 5 = strongly agree) and leave comments on the food innovations.

Following the data analysis method of [11], two authors independently tallied the level of agreement for each item. For the definitions, none received a priori ≥70% agreement. Therefore, the one with the highest level of agreement (eight out of the 13 participants selected either agree or strongly agree) was amended based on the participants’ comments. We identified common words and questions to better formulate the definition based on participants’ comments. We included this definition in round three. For each specific food innovation, only the number of agrees and strongly agrees were counted as agreement. We included the specific food innovations that received a priori ≥70% agreement (ten or more responses were agree or strongly agree) in the round-three questionnaire [33,34]. Due to the great number of innovations (*n* = 9) that received 69.2% agreement, we decided to include them on the round three questionnaire with those ≥70% to clarify their prospective availability.

### 2.5. Round Three

Round three was the consensus building round. Round three had a similar structure to round two, but participants were also asked to rank the likelihood of the food innovation being available to consumers in the next five years with one being the most likely and 15 being the least likely. This was due to there being 15 identified food innovations that reached ≥69% agreement. To analyze round three, similar steps from round two were followed. The 69% threshold for inclusion in the rankings was kept to remain consistent with round two. Two researchers independently counted the level of agreement for each specific food innovation and the new definition. The rankings of the 15 identified food innovations by each expert were summed; those with the lowest score received high levels of availability likelihood and those with high scores had lower levels of availability likelihood [11]. A consensus was determined for the definition if it attained ≥70% agreement [33,34].

## 3. Results

### 3.1. Round One

Based on the respondent-provided definitions, three themes emerged: how food innovations are generated (how), what constitutes a food innovation (what), and the purpose of food innovations (why). Based on these categories, the data were synthesized into three definitions that attempted to capture the meaning of ‘food innovations’ and thirteen themes of food innovations (Table 1). Two independent researchers then repeated the process with the specific food innovations. The experts provided 82 specific food innovations. These innovations were inductively grouped together into themes. Thirteen themes of food innovations emerged (see Table 2). Of the themes, increasing food health had the greatest number (*n* = 13) of mentions from the expert sourced innovations. Then, reducing food waste had the second greatest number of mentions (*n* = 8), followed by food technology (*n* = 7), shorter supply chains (*n* = 7), cropping systems (*n* = 7), food security (*n* = 7), and alternative proteins (*n* = 7). After elimination of repeated and similar innovations, we included 29 specific food innovations on the round-two questionnaire to increase clarity.

### 3.2. Round Two

All 13 experts from the first round responded to the second round. The second-round questionnaire did not contain a ≥70% consensus on any of the definitions. A total of 15 from the original 30 food innovations reached ≥69% consensus (Table 2). We separated them into three groups (high, middle, low) to distinguish the experts’ level of agreement on the food innovations. In group 1, there are 15 food innovations. Plant-based meat alternatives received the highest agreement (100%), followed by supply chain technologies (92.3%), and improving shelf life (84.6%). Natural foods, urban agriculture, and packing innovations (e.g., increased efficiency, receptive packaging) received 76.9% agreement. Nine other innovations received 69.2% agreement. The 15 food innovations were included in round three so that participants could confirm their level of agreement as well as indicate which innovations are most likely to be available to consumers in the next five years. The middle had eight specific food innovations, and the low group had six specific food innovations.

### 3.3. Round Three

In round three, all 13 of the original participants completed the questionnaire. Due to the lack of consensus on the definition in round two, a modified definition based on the participants’ comments was proposed for round three. The proposed amalgamated definition, “food innovations aid in the development, production, or transportation of new food products, processes, or technology to promote human health, food security, or environmental sustainability”, reached a 76.9% level of agreement. We accepted the consensus definition as the agreement was above the established a priori of 70%. We removed three specific food innovations that received a lower than 69.2% level of agreement from the rankings to remain consistent with round two.

The plant-based meat alternatives innovation had the highest level of agreement (100%) and was ranked number one overall (Table 3). Sequentially, personalized nutrition, natural foods, new genetically modified organisms, regenerative agriculture, urban agriculture, packing innovations, alternative flours, improving shelf life, supply chain technologies, improved soil health, and technology for traceability followed. There were some differences in the level of agreement between round two and round three, the greatest of which was supply chain technologies (−23.4%).

## 4. Discussion

### 4.1. Food Innovation Definition

Food innovations are at the forefront of the food system. Our study examined the opinions of food system experts on what food innovations are and then forecasted the most likely available food innovations for consumers within the next five years. Our study proffered a consensus definition for food innovations to advance food security, public health, and environmental sustainability. By advancing these issues, food innovations can balance feeding people, enrich human nutrition, and mitigate the impact of climate change. The food innovation definition proposed reflects the entire agricultural value chain while maintaining breadth for sub-themes across agriculture and food science disciplines. Our definition reflects previous studies about expectations and characteristics of food innovations [35,36,37]. Specifically, the expectations and characteristics of food innovations incorporate increasing human health, promoting environmental sustainability, and increasing production efficiency and efficacy [35,36].

Two of the experts in our study provided comments towards the positive nature of the agreed-upon definition: “*some technologies lead directly to the degradation of the environment while perhaps improving food security*” and “*some foods innovations make food healthier and/or cheaper; others make it less healthy and/or more expensive…aid the environment; some harm the environment*”. Positive bias towards innovations is discussed by [38]; pro-innovation bias has been present in diffusion research for many years. Finding evidence of this based on the solicited opinion of experts is to be expected due to the field of research. It is promising to see how the doubt toward this bias presented by two of the experts demonstrates that the discipline is aware of this bias and that they are cognizant that it can lead to negative consequences. This inherent positive bias towards food innovations may be due to the idea that an item is not innovative if it does not enact a positive change. The inherent idea of an innovation is itself positive, preventing any food-related developments that do not instill positive change from being called a food innovation.

In addition, our study found two factions of experts. One faction supported technology-based solutions to modern agricultural problems while the other supported nature-based solutions. The tension between providing nutrition and protecting the environment is evident in clear factions of experts similar to results found in [39]. The future of the food system relies on the entire agricultural value chain to nourish the global population expansion expected by 2050 [40]. The identified specific food innovations and the definition serve to focus efforts in research and practice. These innovations can help to focus research on consumer acceptance of innovations, which is a desired effect of novel food production. The larger goal of many countries and international organizations is to meet the future food needs of human populations while also preserving the environment [41]. Encouraging sustainable food innovations should be of the utmost importance to the agri-food industry as it navigates the socially and environmentally conscious consumer landscape.

### 4.2. Specific Food Innovations

Our study also pinpointed 12 specific food innovations to which consumers will likely have access in the next five years. Each innovation spans the agricultural value chain. They can directly impact food at the pre-harvest (e.g., new genetically modified organisms and natural foods), post-harvest (e.g., plant-based meat alternatives and alternative flours), distribution (e.g., packing innovations and improving shelf life), and consumption (e.g., personalized nutrition and technology for traceability) stage.

The top two distinct food innovations identified in our study were plant-based meat alternatives and personalized nutrition. Plant-based meat alternatives are prospective as a functional food to encourage human health and agricultural sustainability [3,42,43]. Personalized nutrition has been supported by food scientists to combat endemic nutrition-related chronic diseases [44]. Natural food came in third among the ratings. Several scientists have determined that consumers have begun to prefer natural foods even if they may purchase alternative products [45,46,47]. The differences in consensus between round two and round three may be attributed to the experts receiving additional knowledge through their work with food innovations. In addition, upon further consideration of the specific innovations, the experts may have adjusted their level of agreement.

Based on the rank and consensus percentage, two differing philosophies emerged among the experts. One group was a proponent of technology-based methods to improve the food system while the other supported nature-based methods. This supposed antithesis sheds light on the bifurcation across the agri-food industry. One of the participants provided comments on this dissonance: “*there is a bifurcation with these depending on one’s overarching* [sic] *philosophy about how we should go about improving our food systems.*” Agri-food industry leaders are not immune to the same dichotomy present in consumer opinions [48]. Many consumers who are concerned about their impact on the environment and nutrition are more likely to purchase organic food. However, some consumers who are more concerned with food availability and affordability are more likely to purchase non-organic foods [48]. Even though the issues around food innovations are complex, they can benefit the public.

Therefore, our Delphi study provides an overview of expected food innovations in the next five years. Future developments in the measurement and assessment of food innovations have not been considered, as well as innovations that may progress faster than expected. Some of the specific food innovations are vague to allow further research to pinpoint development and application opportunities for technology and improved varieties. The vagueness of the specific food innovations did lead to some confusion since versions of the innovations may already be available to consumers. This vagueness could have skewed the results towards innovations that may already be available. For example, plant-based meat alternatives already exist; however, new and/or improved alternatives are still in development. Therefore, future research should gain deeper insight into the 12 specific innovation themes. The inclusion of both novel foods and technological processes could be separated to form a more specific forecast of innovations to come.

### 4.3. Implications

#### 4.3.1. Food Innovation Definition

By using the Delphi technique to create consensus definition on food innovations, the definition can be used by both private and public entities to advance their research and development processes. The distinguished features of food innovations can help assess consumer attitudes toward food innovations. Only one participant mentioned that “*consumer acceptance of innovations may vary*”. Understanding consumer acceptance of innovations is vital to the growth and sustainability of the food system. The food innovation definition developed from our study aims to provide clarity for practitioners and policymakers so that work and legislation around food innovations aptly corresponds to the research on food innovations. This could help research more aptly inform policy and practice. Extension specialists could use the definition in education settings as well as to inform producers about upcoming technologies so that they can gauge producers’ interest and include them in the development process.

#### 4.3.2. Specific Food Innovations

The identified food innovations could link food research and the public. Researchers, policymakers, and agri-food industry leaders should conduct research on consumer acceptance of these innovations and further expand on the forecasted food innovations. Agricultural educators, extension agents, and researchers should share comprehensible food innovation information to the public so that food innovations are better understood, especially those highly rated in our study.

Future research should explore the two philosophies identified in the responses of the food system experts in our study. The Diffusion of Innovations Theory built the foundation for innovation adoption, and remains a cornerstone for understanding the agriculture, food, and natural resources industries’ adoption of new technology, products, and ideas [38]. Our study focused on the first stage of the five-stage model (knowledge, persuasion, decision, implementation, and confirmation) [38]. Knowledge emphasizes the necessity of gaining insight into particular innovations, how they work, and why they work [38]. In the knowledge stage of the innovation decision process, researchers, policymakers, and the food industry can frame the messages consumers receive on these innovations to promote adoption [38]. By developing the knowledge of what constitutes a food innovation, creating more awareness of food innovations—and later, how-to knowledge and principles knowledge about them—is possible [38]. Corporate entities that are developing food innovations should begin the process of understanding how the public perceives those innovations.

Our study established a definition of food innovations and expert knowledge of upcoming food innovations. Research on the adoption of innovations is scant without acknowledging the foundation of food innovations. The pinpointed specific food innovations can be used to further research along the five-stage model [38]. Food industry experts, researchers, practitioners, and government officials can use the identified food innovations to capitalize on food innovation research and development. Researchers should investigate the diffusion attributes of imminent food innovations (i.e., relative advantage, trialability, observability, and compatibility, and complexity) as these attributes can significantly influence the rate of adoption [38].

Due to the breadth of some of the food innovations, future research should delve into the food innovation items and assess their possible consumer acceptance. Research on developing specific plant-based meat alternatives, such as cultured meat, fermentation to increase plants’ nutritional value, and microalgal biomass (as well as consumer acceptance of these alternatives), is needed [42]. Personalized nutrition research on consumer acceptance and methods for mitigating the costs of personalized nutrition is vital on account of their potential to address chronic disease and enhance human health [44].

### 4.4. Limitations

This study experienced some limitations. Delphi studies have been used as a prediction tool in a variety of disciplines [17,19,30,33,49]. While the Delphi technique utilizes expert opinions to predict a scenario of the future, forecasting the availability of food innovations is challenging. Therefore, other methods of prediction such as exponential adjustment and regression should be utilized to substantiate this study’s findings. In addition, a Delphi study does not explain how these innovations will emerge; therefore, more research should be conducted on the emergence of future food innovations.

Another issue present in Delphi studies is desirability bias, meaning that experts will select and rate items based on subjective opinions rather than the actual probability that they are likely to happen [24,29]. This is typically diminished with an increase in the number of participating experts, but our study is unlikely to have this bias due to the two factions (technology-based solutions and nature-based solutions) of experts who were present in the study. Bias may also be present in the framing of the questions by the researchers and the bandwagon effect [29]. The wording of the questions needs to be specific in order to solicit accurate responses from the experts [29]. Increasing heterogeneity, such as having participants from relevant but different fields, can also reduce the framing effect since the experts are approaching the question with slightly different backgrounds [29]. The balancing of heterogeneity and homogeneity in a Delphi study is a complex process as both can benefit the study [11,29].

The expert group consisted of 13 participants, despite having reached out to 61 potential participants. The modestly sized expert panel has been supported by several researchers [11,19,32,50,51]. Furthermore, [11] suggests that 10 to 15 homogenous participants may be enough. The homogeneity of the group is evidenced in the separation of 13 themes around food innovations with a minimum of 30% of the participants expressing similar expectations. The exploratory nature of our study, rather than increasing group understanding or gaining group support, aligns with the modestly sized participant panel [11]. Several studies in food research have used similarly sized expert panels to produce consensus documents intended to support future research and practice [50,51].

Due to the modestly sized expert pool, retaining experts was a priority. To accomplish this, the researchers kept the questionnaires as brief as possible. Therefore, it was not possible to assess all relevant aspects of food innovations. One Delphi study is unable to encompass an entire discipline while being highly detailed [17]. Another limitation is the combination of establishing the definition and sourcing upcoming food innovations in our study. This could have influenced the participants as they were identifying the specific food innovations. However, because the same participants were used for both, they would have used their definition of food innovations when creating their list of specific food innovations.

## 5. Conclusions

Food innovations are essential for creating novel nutritious foods, improving agricultural sustainability, and increasing agri-industry market profits. Our study proposes a consensus definition of food innovations and identifies upcoming food innovations that will be available in the next five years. Forecasting future innovations provides an insight into current and developing agri-food research. It can also support the investigation of consumer acceptance of the future innovations. With the new definition developed from our study, researchers and policymakers can clearly guide their research and devote time to focusing on the development of food innovations that align with our definition emphasizing the importance of providing positive outcomes for producers, consumers, and the environment. Incorporating sustainability into the definition highlights the expert focus on increasing the agri-food industry’s environmentalism while supporting an increasing population. The ranked specific food innovations could further connect the agricultural value chain to develop novel nutritious foods and improve agricultural sustainability. Members of the agricultural value chain, from industry specialists to researchers to policy makers, can innovate novel foods that will meet the expectations of sustainability while increasing yield.

## Figures and Tables

**Table 1:** Food innovation definitions and specific food innovation themes.

Definitions	
•Food innovations aid in the development, production, or transportation of diet-centered products, processes, or technology to promote human health and environmental sustainability.	
•Food innovations aid in the development or production of new genetically modified organisms, breeding, cropping, or feeding methods to increase production, food security, and food safety.	
•Food innovations aid in the development, production, or transportation of culturally-appropriate food to increase production and food security.	
**Specific Themes (*n* = 13)**	**Number of Mentions in Theme**
Increasing food health	13
Reducing food waste	8
Food technology	7
Shorter supply chains	7
Cropping systems	7
Food security	7
Alternative proteins	7
New genetically modified organisms	5
Food safety	5
Personalized nutrition	4
Increasing meat quality and quantity	4
Urban agriculture	4
Communications	4

**Table 2:** Specific food innovations likely to be available to consumers by 2027.

Innovations		Count	Consensus Percentage (%)
High	Plant-based meat alternatives	13	100
	Supply chain technologies	12	92.3
	Improving shelf life	11	84.6
	Natural foods	10	76.9
	Urban agriculture	10	76.9
	Packing innovations	10	76.9
	Personalized nutrition	9	69.2
	New genetically modified organisms	9	69.2
	Alternative flours	9	69.2
	Making soil and gut biome connections	9	69.2
	Regenerative agriculture	9	69.2
	Reprocessing food waste	9	69.2
	Improved soil health	9	69.2
	Technology for traceability	9	69.2
	Restaurant digitization	9	69.2
Middle	Nanotechnology	8	61.5
	Using fermentation to increase nutrients	7	53.8
	Nutraceuticals	7	53.8
	Ultraprocessing	7	53.8
	Improved irrigation methods	7	53.8
	Expanding access to government-based poverty alleviation programs	6	46.2
	Improved vaccinations for livestock	6	46.2
	Lower cost cropping systems	5	38.5
Low	Expanding access to company-based poverty alleviation programs	4	30.8
	Phage applications	4	30.8
	3-D printed food	4	30.8
	Insect proteins	3	23
	Urban aquaculture	3	23
	Use of xenobiotics	0	0

**Table 3:** Rank of specific food innovations likely to be available to consumers in the next five years.

Innovations	Round 2 Consensus Percentage (%)	Rank	Round 3 Consensus Percentage (%)	Round 2 to Round 3 Percent Change
Plant-based meat alternatives	100	1	100	0
Personalized nutrition	69.2	2	76.9	+7.7
Natural foods	76.9	3	92.3	+15.4
New genetically modified organisms	69.2	4	76.9	+7.7
Regenerative agriculture	69.2	5	84.6	+15.4
Urban agriculture	76.9	6	76.9	0
Packing innovations	76.9	7	69.2	−7.7
Alternative flours	69.2	8	76.9	+7.7
Improving shelf life	84.6	9	84.6	0
Supply chain technologies	92.3	10	69.2	−23.4
Improved soil health	69.2	11	69.2	0
Technology for traceabilty	69.2	12	69.2	0

## Data Availability

Data is contained within the article.
